# Single Cell Explorer, collaboration-driven tools to leverage large-scale single cell RNA-seq data

**DOI:** 10.1186/s12864-019-6053-y

**Published:** 2019-08-27

**Authors:** Di Feng, Charles E. Whitehurst, Dechao Shan, Jon D. Hill, Yong G. Yue

**Affiliations:** 1 Computational Biology, Boehringer Ingelheim Pharmaceuticals, Inc., 900 Ridgebury Road, Ridgefield, CT 06877 USA; 2 Immunology and Respiratory Disease Research, Boehringer Ingelheim Pharmaceuticals, Inc., 900 Ridgebury Road, Ridgefield, CT 06877 USA; 3 Present Address: Data Science, Camp4 Therapeutics Corp, One Kendall Square, Cambridge, MA 02139 USA

**Keywords:** Single cell, RNA-seq, Pipeline, Transcriptomics, Visualization, Django, D3, Python

## Abstract

**Background:**

Single cell transcriptome sequencing has become an increasingly valuable technology for dissecting complex biology at a resolution impossible with bulk sequencing. However, the gap between the technical expertise required to effectively work with the resultant high dimensional data and the biological expertise required to interpret the results in their biological context remains incompletely addressed by the currently available tools.

**Results:**

Single Cell Explorer is a Python-based web server application we developed to enable computational and experimental scientists to iteratively and collaboratively annotate cell expression phenotypes within a user-friendly and visually appealing platform. These annotations can be modified and shared by multiple users to allow easy collaboration between computational scientists and experimental biologists. Data processing and analytic workflows can be integrated into the system using Jupyter notebooks. The application enables powerful yet accessible features such as the identification of differential gene expression patterns for user-defined cell populations and convenient annotation of cell types using marker genes or differential gene expression patterns. Users are able to produce plots without needing Python or R coding skills. As such, by making single cell RNA-seq data sharing and querying more user-friendly, the software promotes deeper understanding and innovation by research teams applying single cell transcriptomic approaches.

**Conclusions:**

Single cell explorer is a freely-available single cell transcriptomic analysis tool that enables computational and experimental biologists to collaboratively explore, annotate, and share results in a flexible software environment and a centralized database server that supports data portal functionality.

## Background

Rapidly evolving single cell sequencing technologies are enabling researchers to generate data that have the potential to lead to unprecedented biological insight, albeit at the cost of greater complexity of analysis. Open-source, point-and-click, web-based interfaces have become a popular choice to share the analytic results of single cell experiments [1]. More authors now provide R Shiny apps as a solution to share results from specific studies or collections. Other software such as iS-CellR [2] and ASAP [3] provide graphical interfaces for non-R programmers to use specific R packages such as Seurat [4]. However, because of a continuous increase in the creation of experiment types, pipelines and methods, it may be considered impossible to generate a single graphical user interface (GUI) that covers a large number of methods without impairing usability. Many present tools are specialized ‘build to fit’ applications that focus on data exploration of processed data, but do not permit duplication of research findings from raw data. Furthermore, these often are constrained as data exploration tools, rather than being sufficiently full-featured to allow open-ended analysis.

We developed Single Cell Explorer using hybrid approaches, including the application of a Python based programming environment and web app GUI, to enable result sharing and fluid data exploration. The Python based environment was chosen for enhancement of data reproducibility and flexible implementation of a variety of algorithms/workflows, since the integration of Jupyter notebook is becoming increasingly popular in the bioinformatics research community [5]. Single Cell Explorer’s GUI was developed with a focus on easy use and intuitiveness for experimental biologists to explore with minimal training. Single Cell Explorer was developed as a generalized platform for research teams to share and use single cell transcriptome data generated from either pipelines or processed data, with full open access to complex workflows, tools, and methodologies-all behind a simple interface. In contrast to the existing R-based frameworks, Single Cell Explorer will scale to large collections of studies by integrating with modern, performant databases and workflows such as Scanpy [6].

### Implementation

**Fig. 1:**
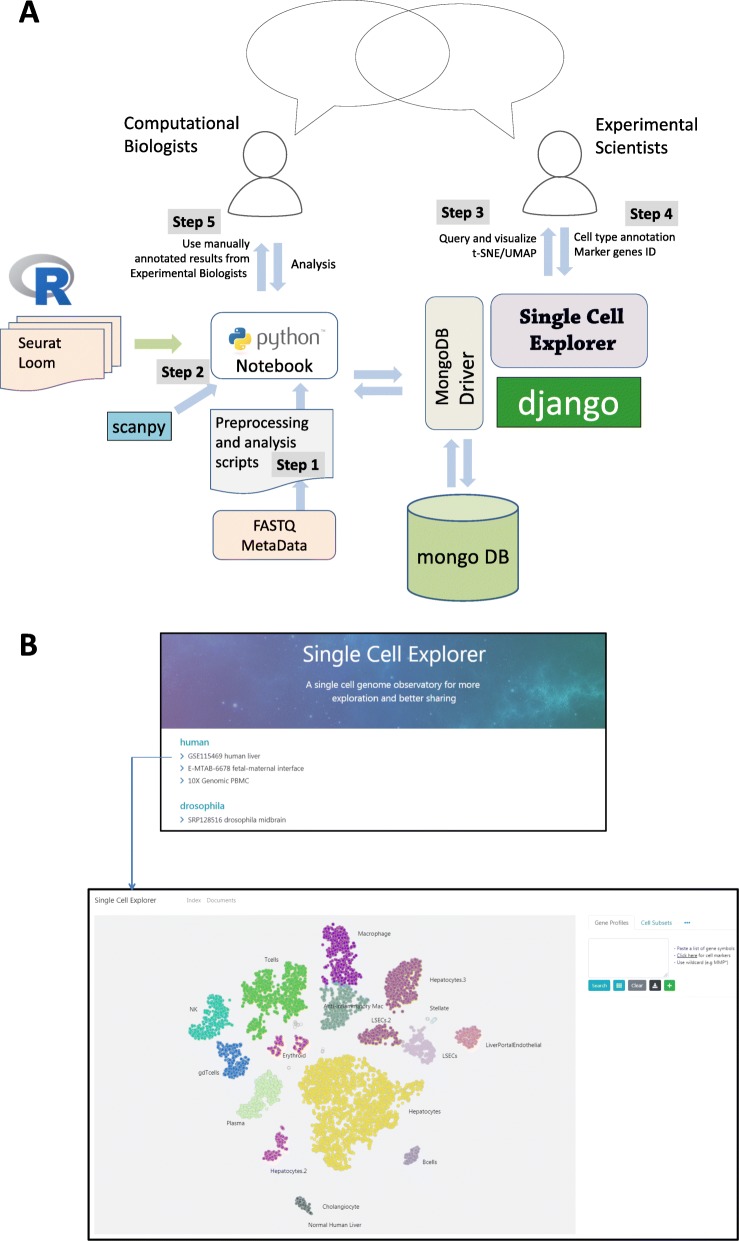
Single Cell Explorer workflow architecture process and component view. **a** Overview of the data process workflow steps for Single Cell Explorer. Step #1: Run pipeline to process FASTQ files using Python wrapper through Jupyter Notebook. Step #2: Quality control of data, generation of 2d representation, and database upload. Step #3: Interactive data analyses and annotation of cell types. Step #4: Recording of annotated results in MongoDB for sharing with all users. Step #5: All results from MongoDB can be accessed directly or via API. **b** A screenshot for Single Cell Explorer data navigator page and a t-SNE map for one dataset

Single Cell Explorer was written using the Python 3.0 programming language, and built with the Django framework. User interactions such as drawing and labeling were written using Javascript. The software is open source and currently available through GitHub at https://github.com/d-feng/SingleCellExplorer. It can be launched by servers which support the Python environment. Python WSGI HTTP Servers for UNIX such as Gunicorn are suggested to support concurrent use of this app. A component view of the system is shown in Fig. 1a, which reveals the integration of analytic pipelines via the Single Cell Explorer database. The steps to use the application are:

1. Raw Data Processing. Initial processing of data is performed using Python Jupyter Notebook or JupyterLab. This step includes read mapping alignment, gene quantitation, and quality control employing Cell Ranger v3.0 (http://10xgenomics.com) to process Chromium single-cell RNA-seq FASTQ data. Alternatively, raw data can be processed using Bash or Nextflow. We provided the Python scripts to integrate the raw data processing pipeline Cell Ranger. The required input files are FASTQ files and the appropriate genome reference files for the relevant organism.
2. Preliminary Analysis in Python Environment. This step runs quality control and dimensionality reduction using the results generated from step 1. The application is agnostic to the method used for dimensionality reduction; both t-Distributed Stochastic Neighbor Embedding (t-SNE) and Uniform Manifold Approximation and Projection **(**UMAP) coordinates have been generated with Seurat or Scanpy methods and used. The principal output of this step includes the filtered cell/gene expression matrix as well as the matrix describing the 2D coordinates of the cells in lower dimensional space. The output is then loaded into a MongoDB database, along with basic metadata about the project to enable project-level queries.
3. Collaborative Analysis through Web and API. After the data has been loaded, the web front end enables users to visualize and query downstream analytic results through interaction with the lower-dimensional map of the cells. This step relies on JavaScript, SVG (Scalable Vector Graphics), HTML5 (Hypertext Markup Language), and CSS (Cascading Style Sheet) to enable an interface which is highly responsive and scales well. In addition to basic data exploration, cell type annotations can be captured by users and stored. For highly customized analyses, API (Application Programming Interface) functions enable bioinformaticians to work directly with the database.

## Results and discussion

### Single cell RNA-seq data processing and analysis

As an example of the utility of Single Cell Explorer, a test run was performed on a publicly available dataset of human peripheral blood mononuclear cells (PBMCs) from (https://support.10xgenomics.com/single-cell-gene-expression/datasets). We showed the case of using a Jupyter notebook to drive a 10X genomics based cell processing pipeline. The Cell Ranger pipeline can be started using runCellrangerProcess, a function in the notebook, and is followed by the Scanpy analytic workflow in the same Jupyter notebook for quality control and dimensionality reduction. For Jupyter notebook users, we provided scpipeline, which is a python script for a helper function that runs Cell Ranger and loads the result to MongoDB. The project metadata, cell/gene expression matrix, normalized data, and results of the 2D cell mapping will be uploaded by the notebook to the MongoDB instance.

### Interactive tertiary results access from web page

**Fig. 2:**
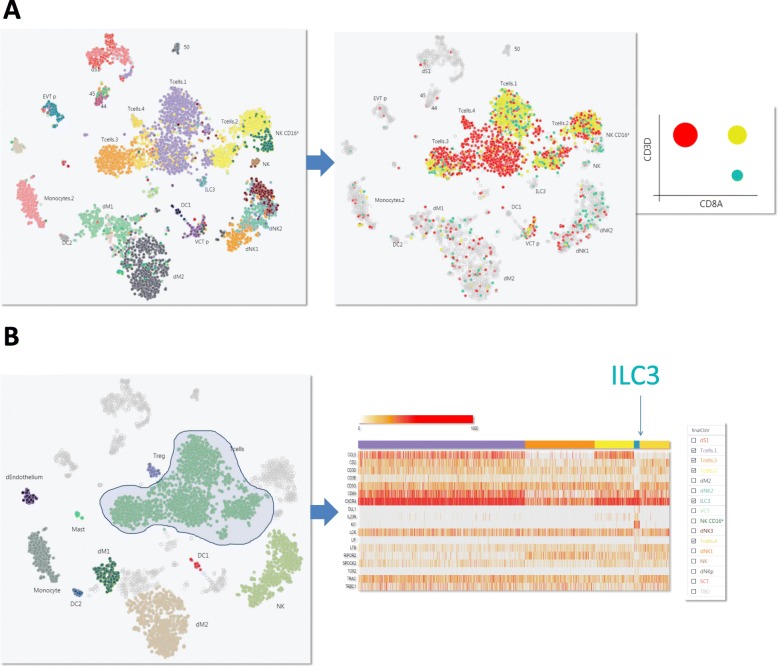
Interactive FeaturePlot. **a** A t-SNE and UMAP representation from first-trimester placentas with matched maternal blood and decidual cells. Individual pre-labeled cell types are painted in different colors. The function of painting two genes (CD8A and CD3D) highlights the location of CD8 T cell clusters. A 2D plot of circles indicates the proportion of the single positive and double positive cells. **b** To query a list of genes, a heatmap can be generated after freehand selection of cells of interest. ILC3 cells can be identified using markers including KIT and DLL1

For high-dimensional single cell data, lower dimensional representations such as t-SNE or UMAP are necessary to interact with the data and to easily observe broad relationships between cells (Fig. 1b). Single Cell Explorer supports all types of low-dimensional representation [7]. Here we showed the re-analysis of single cell RNA-seq data for cells from the early human maternal-fetal interface [8]. The multiple types of metadata, including cell types, cluster information, and sample information such as tissue, donor, and any other clinical features, can be overlaid on the feature plot. The user interface provides a simple gene expression search function for each feature plot. A box plot of normalized counts and the percentage of cells with positive expression (counts > 1) will be shown for querying single gene expression. The interface also supports queries for two genes simultaneously, with the gene expression pattern painted with different colors. If multiple genes were searched, users are able to generate a heatmap using normalized counts or z score (Fig. 2b).

### Cell type identification and annotation

**Fig. 3:**
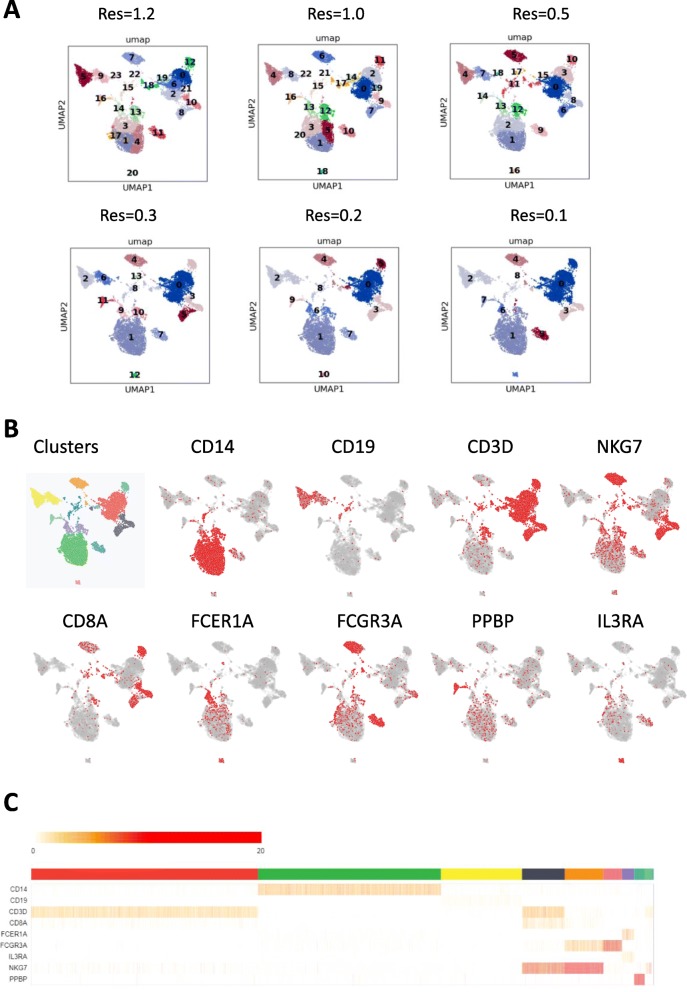
Understanding single cell clustering results. **a** UMAP of human normal PBMC with various clustering results using different resolution parameters by the leiden algorithm (scanpy.api.tl.leiden function). **b** Feature plot of cells which are positive for each individual marker gene. **c** A heatmap of marker gene expression within each cluster defined by leiden algorithm

A key challenge for analyzing single cell RNA-seq data is choosing an optimal cell clustering parameter that best delineates the key cell sub types. For example, the data plot in Fig. 3a shows various clustering results using different resolution parameters by the leiden algorithm (scanpy.api.tl.leiden function). Note that the default resolution value of 1 produces more clusters of cell types than the key major immune cell types we typically like to identify in our routine expression analyses. It remains quite difficult to determine which resolution value to use for optimal future analyses.

**Fig. 4:**
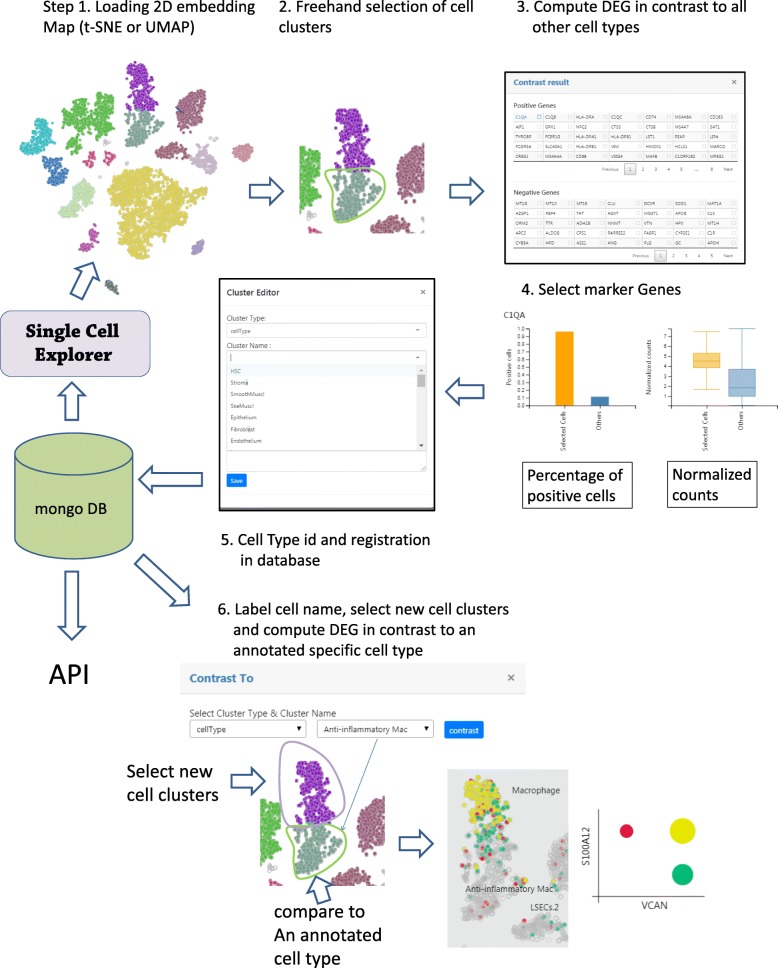
Cell type and feature discovery. Step #1: Load 2D embedding map. Step #2 Use a freehand tool to select the cells of interest. Step #3: Compare the differentially expressed genes of selected cells with all unselected cells. Step #4: Interactively visualize gene expression levels using the resulting table. Step #5: Record cell types and marker genes for future reference. Step #6: Position the newly-labelled cells on the map and compare with other specific cell types

Single Cell Explorer enables users to examine expression of key cell type markers in UMAP (e.g. CD14 (Monocyte 1), CD19 (B cells), CD3D (T cells), NKG7 (NK cells), FCGR3A (Monocyte 2), CD8A (CD8 T cells), FCER1A (DC), IL3RA (Plasmacytoid Dendritic Cells), PPBP (Platelets)). Users can match the marker gene expression with the cell cluster identification number using the heatmap function (Fig. 3b). In this case, it is most reasonable to use a value of 0.2 for the resolution setting. When cell type composition is unclear or cell marker information remains unknown, differentially expressed genes can be obtained first. Then these newly identified cell markers or domain knowledge can be used to annotate cell clusters in the 2D map (Fig. 4). First, the user can click and draw circles to select the cell cluster of interest. Next, a contrast function is executed for computing differentially-expressed genes between selected clusters and all other cells. The non-parametric Wilcoxon rank sum test is chosen as the default method due to fast execution time and comparable performance among other algorithms [9]. The computed results will be shown as a table with *p*-value-ranked genes increased or decreased in expression. The user can click the differentially-expressed genes which will be distinguished by their color on the t-SNE plot. The user can name the cell type by choosing cell type name from a list (to enforce controlled vocabulary), or add new names that do not exist in the database. The other statistical methods can be applied in Jupyter Notebook or R Studio. This capability not only allows users to delineate and explore potential new cell subset types, but also enables single cell data sets to be viewed from different dimensions beyond pre-set or pre-conceived cell marker paradigms, potentially fostering innovative viewpoints and new hypotheses.

### Database and API

The annotated data will be displayed in the web application. The following Python API functions (Table 1) were designed to retrieve data from the Single Cell Explorer database. The map id is unique for each map. clusterName is the annotated cell type. clusterType includes cell type and other information such as donor, samples, and shared nearest neighbor’s cluster id. The API can be used to compute differentially expressed genes, or for other bioinformatics analysis between annotated cells within samples as well as across different samples, using Jupyter notebook.

**Table 1:** API function to retrieve data from database

Name	Function
getAllClstrsByClstrsType	retrieve a table of cell barcodes and annotated cell types in a specific map
getNormalizedGeneExpr	get normalized counts matrix for genes of interest from specific cell types in a specific map
getAllNormalizedGeneExpr	get full normalized gene counts matrix from specific cell types in specific map
getMarkGenesByMapidAndClusterType	get annotated marker genes
getMaps	get meta data from a specific map
exportAllClstrsByClstrsType	export the cell barcodes and annotated cell types of the cells from a specific map into a csv file

### Comparison to other software

To our best knowledge, no other single cell sequencing software currently provides reanalysis capabilities that include drawing, annotation, saving the results in a database, and integration with Jupyter notebook for more complex analyses. Cellxgene [10] is a Python-based interactive data visualization tool for single-cell transcriptomic datasets, but it focuses on well curated single data sets without comprehensive database support. It will show data objects, but for each data object, it requires a new instance or independent port. Since the time required to load a data object file (h5ad file) is long, it is difficult to use Cellxgene as a data portal. On the other hand, our application is built for concurrent users to explore an unlimited number of datasets, due to our implementation of MongoDB. Also, in contrast to canvas, which is suitable for displaying large numbers of cells, we use SVG to allow faster information accessibility and better interactive performance for data sets with fewer cells.

## Conclusions

We developed Single Cell Explorer, a Python-based platform which promotes a collaborative data sharing experience for single cell transcriptomic data. It balances a high degree of automation integration with open source tool sets and a visually-attractive end user experience. For a genomics core lab, a complete workflow analysis empowered with automation allows experimental scientists ease in previewing their results, quickly promoting faster cycles of hypotheses building and experimental innovation. Computational biologists can also analyze data sets using different methods to generate 2D plots of data findings to load and share with research teams. Using the web app coupled with a centralized MongoDB server, team members can label and share findings to promote further cycles of inquiry and hypothesis generation.

## Data Availability

**Project name:** Single Cell Explorer **Project home Page:** http://www.singlecellexplorer.org or http://54.159.6.229:80/ Home page includes a software manual with videos, a demo, and code. **Repository:** https://github.com/d-feng/SingleCellExplorer **Demo software:** http://54.159.6.229:8000/ **Python notebook:** http://54.159.6.229:8001/ **Operating Systems:** Server: Linux, Client: Platform independent **Programming Language:** Python, Javascript **Other Requirements:** Python3.6, Mongodb 3.6, Django 2.0 or above, scanpy, scipy, numpy, pymongo, scipy, pandas, numpy, subprocess, sklearn, bootstrap 4, jquery 3, d3.v4.js, optional softwares to enhance the function: cellranger 3.0, jupyter notebook, gunicorn **Recommended Hardware**: CPU: 3 GHz above, Memory: 8G above **License:** GNU GPL version 3 **Any Restrictions for use by non-academics:** None.

## Abbreviations

API: Application programming interface
GUI: Graphical user interface
HTML5: Hypertext Markup Language 5
PBMCs: Peripheral blood mononuclear cells
SVG: Scalable Vector Graphics
t-SNE: t-Distributed Stochastic Neighbor Embedding
UMAP: Uniform Manifold Approximation and Projection
